# Supplementary material for: Inhibitory Control and the Structural Parcelation of the Right Inferior Frontal Gyrus
Source: Front Hum Neurosci. 2022 Feb 24;16:787079. doi: 10.3389/fnhum.2022.787079 (PMC8907402; doi:10.3389/fnhum.2022.787079)
Supplement: Supplementary file 1 [file Data_Sheet_1.docx]

Supplementary Material

# Supplementary Note

To further test the robustness of the dorsal pars opercularis-supplementary motor area complex connection and the stop signal task go reaction time and stopping accuracy, we reran the linear regression model in the main analysis by controlling for sex and age instead of the global fractional anisotropy value. The p-value was set to 0.05 /4 comparisons = .0125 to adjust for multiple comparisons. The results were in line with the results from our main analysis, showing a consistent association to the stop signal go reaction time and stopping accuracy, independent of age and sex (Supplementary Table 1).

**Supplementary Table 1.** *Summary of Robustness Analyses (N =20)*

|  | *B* | *SE B* | *β* | *t* | *P* |
| --- | --- | --- | --- | --- | --- |
| ***SST go reaction time (***R^2^ = .390, adjusted R^2^ = .282, F =3.623, p = .035) | | | | | |
| Intercept | 46.571 | 235.448 |  | .198 | .846 |
| Age | -1.378 | 3.227 | -.084 | -.427 | .675 |
| dOp-SMAc | 2463.243 | 860.776 | .908 | .908 | .011 |
| vOp-SMAc | -1246.864 | 968.076 | -.419 | -.419 | .215 |
| ***SST stop accuracy, % (***R^2^ = .430, adjusted R^2^ = .330, F = 4.278, p =.020) | | | | | |
| Intercept | 16.618 | 13.710 |  | 1.212 | .242 |
| Age | -.107 | .188 | -.109 | -.571 | .575 |
| dOp-SMAc | 114.105 | 50.121 | -.882 | 2.875 | .011 |
| vOp-SMAc | -56.009 | 56.369 | -.312 | -.994 | .334 |
| ***SST go reaction time (***R^2^ = .386, adjusted R^2^ = .278, F = 3.556, p = .036) | | | | | |
| Intercept | 17.748 | 244.878 |  | .072 | .943 |
| Sex | 6.920 | 24.944 | .054 | .277 | .785 |
| dOp-SMAc | 2514.343 | 865.649 | .927 | 2.905 | .010 |
| vOp-SMAc | -1327.196 | 947.104 | -.446 | -1.401 | .179 |
| ***SST stop accuracy, % (***R^2^ = .454, adjusted R^2^ = .358, F = 4.717, p = .014) | | | | | |
| Intercept | 11.784 | 13.912 |  | .847 | .409 |
| Sex | 1.480 | 1.417 | .193 | 1.044 | .311 |
| dOp-SMAc | 151.408 | 49.178 | .927 | 3.079 | .007 |
| vOp-SMAc | -60.490 | 53.805 | -.337 | -1.124 | .227 |

*Note.* SST = stop signal task; dOp = dorsal pars opercularis, vOp = ventral pars opercularis, SMAc = supplementary motor area complex. All predictors had tolerance >.1 and variation inflation factor < .3

# Supplementary Note

We computed pair-wise t-tests between the node degrees for each subregion in the right inferior frontal gyrus and the parcellated pars opercularis (i.e., dorsal and ventral right pars opercularis) using the regions that passed the 80% detection threshold. That is, the interconnections that showed a reliable connection in at least 80% of the individuals. Similar results to those reported in the main text were found. For the terminating connections, the pars opercularis exhibited a lower node degree compared to the pars triangularis (t (29) = -10.77, p < .001) and the pars orbitalis (t (29) = -15.27, p <.001), and the pars triangularis showed lower node degree compared to the pars orbitalis (t(29) = 10.30, p <.001). For the passing connections, the pars opercularis had a lower node degree compared to pars triangularis (t (29) = -25.44, p <.001) and the pars orbitalis (t (29) = -70.76, p <.001). Similarly, the pars triangularis showed lower node degree compared to pars orbitalis (t (29) = -41.78, p <.001). For the parcellated pars opercularis, the dorsal pars opercularis showed a higher node degree for both terminating (t (29) = 9.97, p <.001) and passing connections (t (29) = 8.56, p <.001) compared to the ventral pars opercularis.


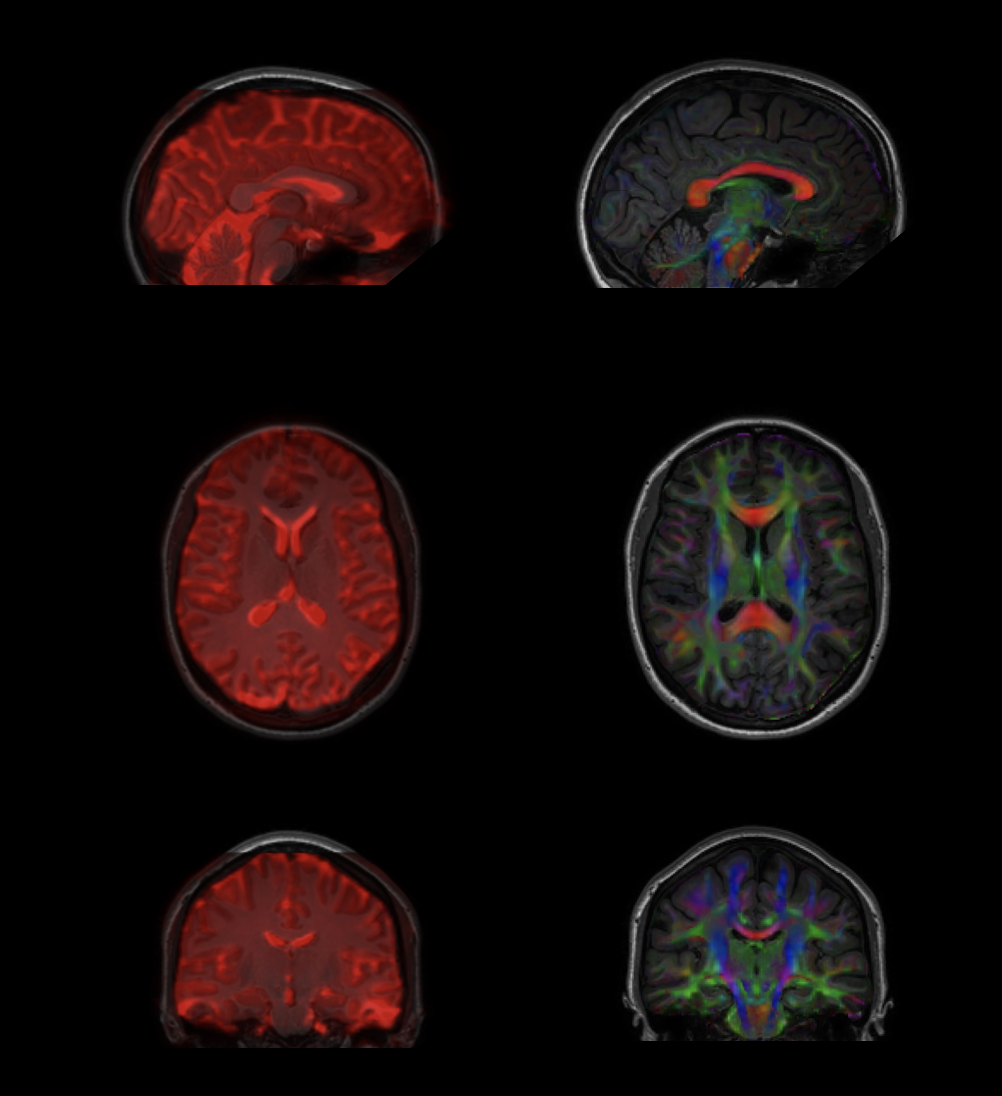

**Supplementary Figure 1.** Pre- and post-correction. The raw B0 image overlaid upon the T1 image before the correction procedure (left). The preprocessing steps yielded corrected diffusion data that was resampled to the size of the T1 image (right), which was further utilized for the whole brain connectivity analysis. Color coding: red = left-right, blue = superior-inferior, green = anterior-posterior.


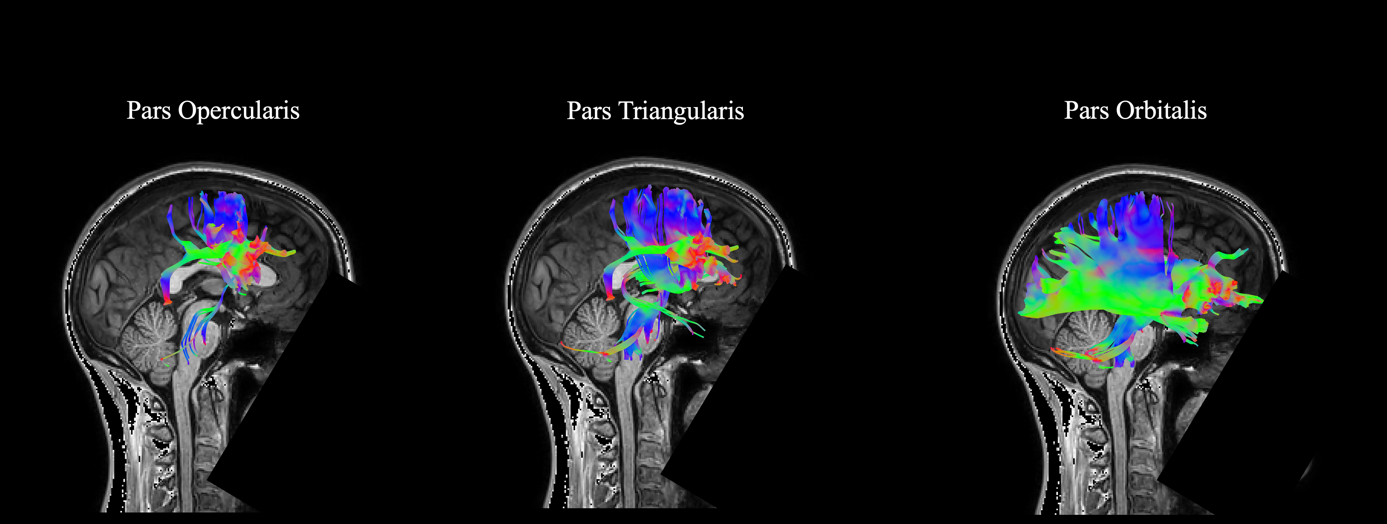
**Supplementary Figure 2.** Reconstructed streamlines from a sample participant. Passing streamlines seeding from the pars opercularis (left), the pars triangularis (middle), and the pars orbitalis (right). Color coding for the orientation of the reconstructed tracts: red = left-right, blue = superior-inferior, green = anterior-posterior.
